# Effect of Accommodating Elastic Bands on Mechanical Power Output during Back Squats

**DOI:** 10.3390/sports6040151

**Published:** 2018-11-22

**Authors:** Takafumi Kubo, Kuniaki Hirayama, Nobuhiro Nakamura, Mitsuru Higuchi

**Affiliations:** 1 Graduate School of Sport Sciences, Waseda University, Tokorozawa, Saitama 359-1192, Japan; t.k-52-waseda@ruri.waseda.jp (T.K.); nobu0902@toki.waseda.jp (N.N.); 2 Faculty of Sport Sciences, Waseda University, Tokorozawa, Saitama 359-1192, Japan; k.hirayama@waseda.jp

**Keywords:** back squats, variable resistance exercise, deceleration subphase, acceleration subphase, power exercise

## Abstract

The aim of this study was to investigate whether accommodating elastic bands with barbell back squats (BSQ) increase muscular force during the deceleration subphase. Ten healthy men (mean ± standard deviation: Age: 23 ± 2 years; height: 170.5 ± 3.7 cm; mass: 66.7 ± 5.4 kg; and BSQ one repetition maximum (RM): 105 ± 23.1 kg; BSQ 1RM/body mass: 1.6 ± 0.3) were recruited for this study. The subjects performed band-resisted parallel BSQ (accommodating elastic bands each sides of barbell) with five band conditions in random order. The duration of the deceleration subphase, mean mechanical power, and the force and velocity during the acceleration and deceleration subphases were calculated. BSQ with elastic bands elicited greater mechanical power output, velocity, and force during the deceleration subphase, in contrast to that elicited with traditional free weight (*p* < 0.05). BSQ with elastic bands also elicited greater mechanical power output and velocity during the acceleration subphase. However, the force output during the acceleration subphase using an elastic band was lesser than that using a traditional free weight (*p* < 0.05). This study suggests that BSQ with elastic band elicit greater power output during the acceleration and deceleration subphases.

## 1. Introduction

Lower body power is related to athletic performance (e.g., sprinting) [1,2]. Rapid movements against loads lead to the development of lower-body power. The mechanical power output during back squats (BSQ) has been shown to be the highest at moderate load (56% 1RM) [3]. However, BSQ with a moderate load (45% 1RM) that cause rapid movements include a “deceleration subphase” at the end of the concentric phase (about 30% of the concentric phase) [4]. The acceleration reaches less than zero as a result of the barbell decelerating at the end of the concentric phase [5]. In other words, as force is equal to mass multiplied by acceleration, muscular force reaches less than that in the quiet standing position during the deceleration subphase. Therefore, increasing muscular force during the deceleration subphase may be necessary to stimulate muscles throughout the full range of motion during a power exercise [6].

Weightlifting movements, ballistic exercise (e.g., jump squat), and variable resistance exercise (VRE) are thought to be exercises that increase muscular force during the deceleration subphase and are used by many strength and conditioning coaches in their fields [7,8,9,10,11,12,13,14]. However, the weightlifting movement is difficult to learn, in contrast to other exercises, and ballistic exercise might cause high mechanical stress to the athlete’s spine and knee [15]. In contrast, VRE might be a little easier to perform and is safer, in comparison to ballistic exercises.

However, to the best of our knowledge, there is no evidence to suggest that VRE could increase muscular force during the deceleration subphase. The optimal resistance of elastic bands during VRE to increase muscular force during the deceleration subphase is also unclear. Therefore, the aim of this study was to investigate whether VRE increase muscular force during the deceleration subphase and to determine the optimal additional resistance of elastic bands. The band tension decreases the first half of the concentric phase and increases at the end of the concentric phase. Based on this characteristic, we hypothesized that as band resistance increases, the muscular force during the deceleration subphase might increase during BSQ.

## 2. Materials and Methods

### 2.1. Experimental Design

**Figure 1:**
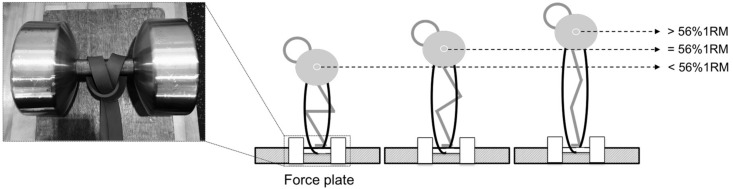
Elastic band settings during band-resisted back squat. The elastic bands were tied on each side of the barbell with “choked” squat attachment [17]. The height of the subjects and the elastic band resistance were adjusted using plyometric boxes below the force plate as loads equal to 56% 1RM at the middle point of the concentric phase.

To investigate the effects of a wide range of band resistance, subjects performed band-resisted parallel BSQ with five different band conditions in a random order, with 56% of 1 repetition maximum (1RM) on a force plate. The BSQ depth was confirmed visually, with the thigh being parallel to the floor. Two elastic bands (100% rubber) are tied in with the dumbbell to each side of barbell (Figure 1). A 56% 1RM elicits greater mechanical power output during BSQ, which was in line with previous research [3]. Band resistance 0% (B0) came from weight plates only. Band resistance 20% (B20) was performed such that the resultant total resistance (100%) came 80% from weight plates and 20% from band resistance. Band resistance 40% (B40), 60% (B60), and 80% (B80) were performed using the same concept. Barbell velocity during BSQ was calculated using linear position transducer (LPT) (PT101; Celesco, Chatsworth, UK). The duration of the deceleration subphase, the mean mechanical power, and the force and velocity during the acceleration and deceleration subphases were calculated using the data of the force plate and linear position transducer.

### 2.2. Subjects

Ten healthy men (mean ± standard deviation: Age: 23 ± 2 years; height: 170.5 ± 3.7 cm; mass: 66.7 ± 5.4 kg; BSQ 1RM: 105 ± 23.1 kg; and BSQ 1RM/body mass: 1.6 ± 0.3) who had performed resistance training for at least 1 year were recruited for this study. The subjects were familiar with lifting with maximal intent in competitions and daily training. All subjects were informed of the experimental procedure, potential risks, and purpose of this study, and a signed informed consent document was obtained before the investigation. The study was approved by a local Ethical Review Committee on Research with Human Subjects.

### 2.3. Procedures

The subjects attended two sessions on nonconsecutive days, separated by at least 48 h: A 1RM session and measurements of height, body mass, and barbell position were conducted on the first day, and a testing session was conducted on the second day. The 1RM session was conducted according to the National Strength and Conditioning Association guidelines [16]. The top (standing) and bottom (parallel) positions of the barbell during BSQ were measured using a tape measure in units of 0.5 cm. The bottom position during measurements and testing session was confirmed visually, with the thigh being parallel to the floor. On the second day, the subjects were warmed up for 5 min on a cycle ergometer with a workload of 60 W. The subjects then performed a warm-up set with 50% 1RM for 10 repetitions and 56% 1RM including 20% elastic bands resistance for 5 repetitions. After the warm-up, the subjects performed 3 repetitions of parallel BSQ with B0, B20, B40, B60, and B80 on a force plate (PH-6110A; DKH Co., Ltd., Tokyo, Japan) in a random order. The eccentric phase was controlled based on the duration of the phase (about 3 s), and the concentric phase was performed by instructing the subject to lift the barbell as fast and hard as possible. The bar placement was high, and the foot stance was at shoulder width [16]. The subjects were instructed to not to try to jump, lift their heels, or pause at the bottom. If these requirements were not met, the trial was repeated again. A 5 min rest was taken between trials. The repetition in which maximal velocity was observed was adopted as the force-time data.

### 2.4. Measurements

Force signals from the force plate were collected at 1000 Hz using an A/D converter (Powerlab; ADInstruments, Castle Hill, Australia). Chart version 5.0 for Windows (ADInstruments) was used for recording data. MATLAB version 2017a (MathWorks, Natick, MA, USA) was used for analyzing data. Raw force-time data were used to analyze the data.

Barbell velocities during BSQ were calculated from displacement data through differentiation data. Barbell displacement data was measured using LPT (PT101; Celesco, Chatsworth, UK). Signals from LPT were filtered by a 10-Hz low-pass zero-phase-lag finite impulse response filter. Four elastic band (S&C Band; S&C Corporation, Tokyo, Japan) resistances were determined in line with current recommendations [17]. Band resistance during BSQ was applied at the middle point of the concentric phase based on barbell position measured on the first day. For example, if the top position was 170 cm and the bottom position was 90 cm, the middle point of the concentric phase was 130 cm ((170 – 90)/2 + 90). Therefore, 130 cm was substituted for the equation of band resistance (Table 1). The resistances from the two elastic bands are the following: Green = 46.8 kg, blue = 24.4 kg, red = 18.1 kg, and purple = 10.4 kg. The equations of band resistance and R^2^ values from this study are shown in Table 1.

**Figure 2:**
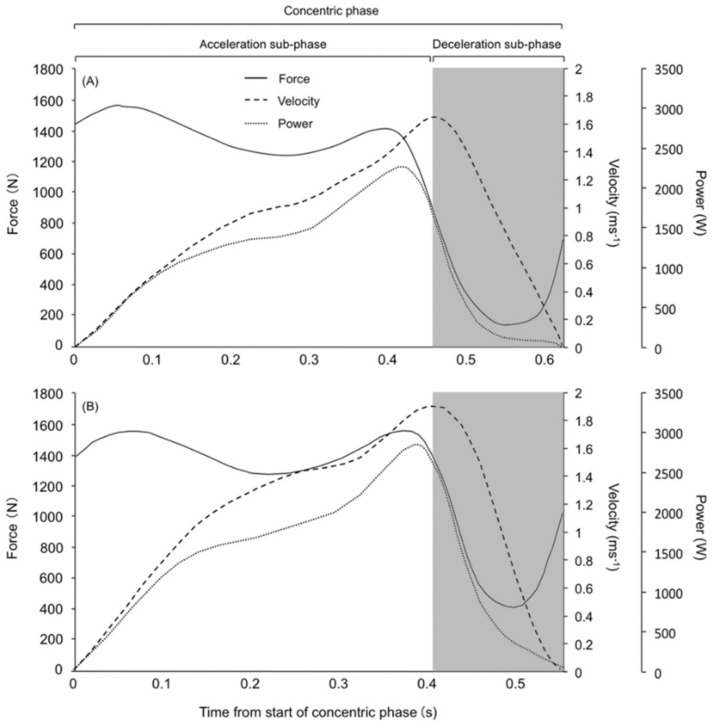
Typical example of a force-time curve during the concentric phase with B0 (**A**) and B80 (**B**). The white area represents the acceleration subphase, and the gray area represents the deceleration subphase.

The start of the concentric phase was defined as the point at which the velocity became positive, that is, a velocity above zero. The end of the concentric phase was defined as the point at which the velocity returns to zero. The point at which the acceleration became lower than zero was considered to be the start of the deceleration subphase [4,5]. The start of the concentric phase to the start of the deceleration subphase was considered as part of the “acceleration subphase.” The absolute duration of the deceleration subphase was termed the “deceleration subphase duration,” and the ratio of duration of the deceleration subphase to concentric phase was termed “relative deceleration subphase duration.” The definition of each phase during BSQ is shown in Figure 2.

### 2.5. Statistical Analyses

Descriptive data were expressed as mean ± standard deviation. If data did not follow normal distribution, one-way repeated measures analyses of variance (ANOVA) and a Bonferroni post hoc test were used to determine whether significant differences existed for duration and velocity. If data followed normal distribution, two-way repeated measures ANOVA (2 phases × 5 band conditions) and a Bonferroni post hoc test were used to determine whether significant differences existed for mechanical force and power. The statistical significance for all analyses was set at *p* < 0.05. Cohen’s d provided the measure of the magnitude of the differences among the no-band (B0) and band conditions (B20, B40, B60, and B80) (< 0.20; small, 0.20–0.50; medium, 0.50–0.80; large, 0.80–1.30; or very large, >1.30) [18]. All statistical analyses were performed using IBM SPSS Statistics version 24 (IBM Corp., Armonk, NY, USA).

## 3. Results

### 3.1. Force

**Figure 3:**
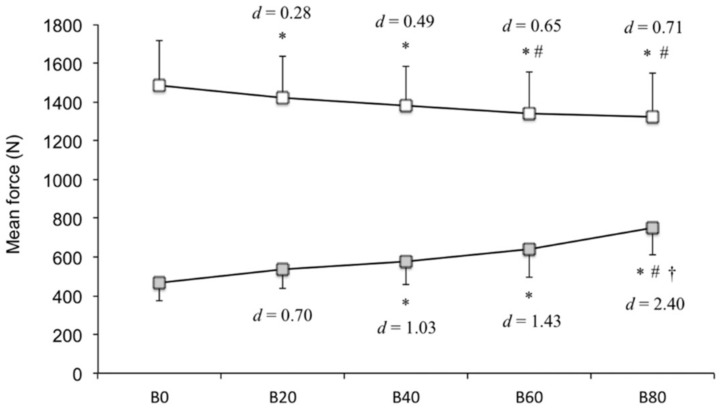
Mean force during the acceleration and deceleration subphases (N). The white square represents the acceleration subphase, and the gray square represents the deceleration subphase. Statistically significant, compared with * B0, ^#^ B20, and ^†^ B40 (post hoc test, *p* < 0.05).

The results of the two-way ANOVA (*p* < 0.05) were as follows: Two phases (F[1, 9 = 230.95], *p* < 0.05), five band conditions (F[4, 36 = 3.66], *p* < 0.05), and two phases × five band conditions interaction (F[4, 36 = 44.34], *p* < 0.05). The follow-up Bonferroni post hoc test revealed that B0 elicited greater mean force during the acceleration subphase compared with that by B20, B40, B60, and B80; B20 elicited greater mean force compared with that by B60 and B80 (*p* < 0.05). The mean force during the deceleration subphase was significantly different according to load (two-way ANOVA, *p* < 0.05). The follow-up Bonferroni post hoc test revealed that it was significantly higher with B40, B60, and B80, in contrast to that with B0; B80 elicited a greater mean force compared with that by B20 and B40 (*p* < 0.05). Summarizing the above, as the band tension increased, the mean force during the acceleration subphase (B20 [*d* = 0.28], B40 [*d* = 0.49], B60 [*d* = 0.65], and B80 [*d* = 0.71]) decreased, whereas, that in the deceleration subphase, (B20 [*d* = 0.70], B40 [*d* = 1.03], B60 [*d* = 1.43], and B80 [*d* = 2.40]) increased (Figure 3).

### 3.2. Duration

**Figure 4:**
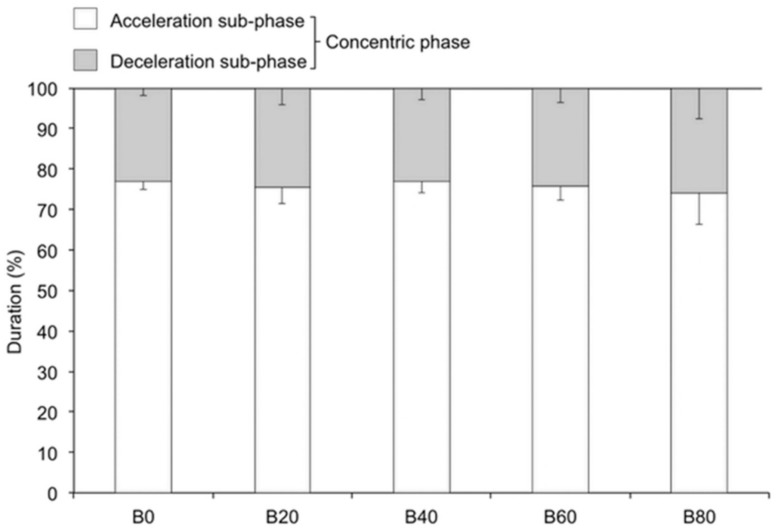
Relative duration of the acceleration and deceleration subphases (%).

The one-way ANOVA (*p* < 0.05) and follow-up Bonferroni post hoc test revealed that the duration of the concentric phase was significantly shorter with B20, B40, B60, and B80, in contrast to that with B0; the duration of the concentric phase with B40 was significantly shorter than that with B20; and the duration of the concentric phase with B60 was significantly shorter than that with B40 (*p* < 0.05). The deceleration subphase duration was significantly shorter with B40 and B60, in contrast to that with B0; the deceleration subphase duration with B60 was significantly shorter than that with B20. Summarizing the above, the relative duration of deceleration subphase was not significantly different among loads (Figure 4).

### 3.3. Velocity

**Figure 5:**
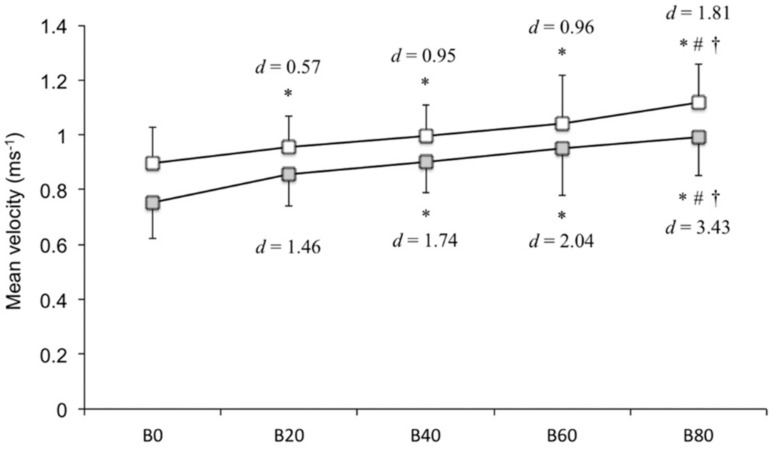
Mean velocity during the acceleration and deceleration subphases (ms^−1^). The white square represents the acceleration subphase, and the gray square represents the deceleration subphase. Statistically significant, compared with * B0, ^#^ B20, and ^†^ B40 (post hoc test, *p* < 0.05).

The one-way ANOVA (*p* < 0.05) and follow-up Bonferroni post hoc test revealed that B20, B40, B60, and B80 elicited higher mean velocity during the acceleration subphase compared with that by B0; B80 elicited higher mean velocity compared with that by B20 and B40 (*p* < 0.05). The mean velocity during the deceleration subphase was significantly different according to load (two-way ANOVA, *p* < 0.05). The follow-up Bonferroni post hoc test revealed that it was significantly higher with B40, B60, and B80, in contrast to that with B0; B80 elicited higher mean velocity compared with that by B20 and B40 (*p* < 0.05). Summarizing the above, as the band tension increased, the mean velocity during the acceleration (B20 [*d* = 0.57], B40 [*d* = 0.95], B60 [*d* = 0.96], and B80 [*d* = 1.81]) and deceleration subphases (B20 [*d* = 1.46], B40 [*d* = 1.74], B60 [*d* = 2.04], and B80 [*d* = 3.43]) increased (Figure 5).

### 3.4. Mechanical Power

**Figure 6:**
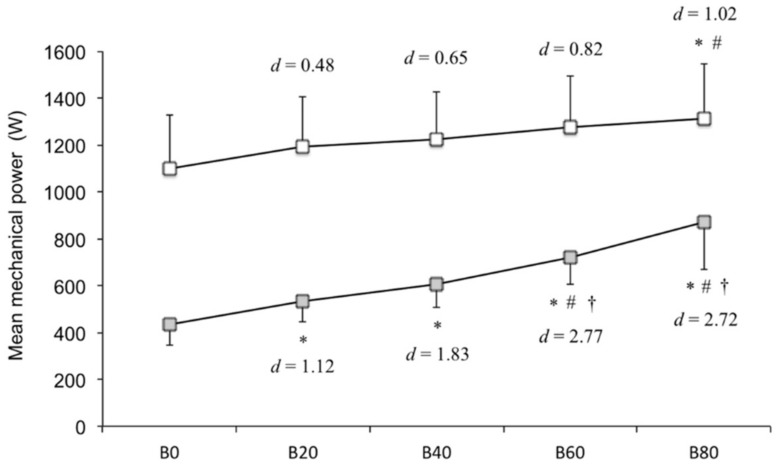
Mean mechanical power output during the acceleration and deceleration subphases (W). The white square represents the acceleration subphase, and the gray square represents the deceleration subphase. Statistically significant, compared with * B0, ^#^ B20, and ^†^ B40 (post hoc test, *p* < 0.05).

The results of the two-way ANOVA (*p* < 0.05) were as follows: Two phases (F[1, 9 = 138.07], *p* < 0.05), five band conditions (F[4, 36 = 43.22], *p* < 0.05), and two phases × five band conditions interaction (F[4, 36 = 8.69], *p* < 0.05). The follow-up Bonferroni post hoc test revealed that B80 elicited greater mean mechanical power output during the acceleration subphase compared with that by B0 and B20. Mean mechanical power during the deceleration subphase was significantly different according to load (two-way ANOVA, *p* < 0.05). The follow-up Bonferroni post hoc test revealed it to be significantly greater with B20, B40, B60, and B80 compared with that with B0; B60 and B80 elicited greater mean mechanical power output than B20 and B40 (*p* < 0.05). Summarizing the above, as the band tension increased, mean mechanical power output during the acceleration (B20 [*d* = 0.48], B40 [*d* = 0.65], B60 [*d* = 0.82], and B80 [*d* = 1.02]) and deceleration subphases (B20 [*d* = 1.12], B40 [*d* = 1.83], B60 [*d* = 2.77], and B80 [*d* = 2.72]) increased (Figure 6).

## 4. Discussion

The study determined the acute effect of different band resistance on the deceleration subphase during BSQ. There was no significant difference in the ratio of the duration of the deceleration subphase to the concentric phase among all band conditions. Meanwhile, as the band resistance increased, the mean mechanical power output (barbell mass multiplied by barbell kinematics, although not kinematics of center of mass), velocity, and force during the deceleration subphase increased. These results supported our hypothesis that, as band resistance increases, the muscular force during the deceleration subphase might increase during BSQ. However, this study also revealed that, as band resistance increased, the mean force during the acceleration subphase decreased. Considering these results, although BSQ with elastic bands decreased the mean force during the acceleration subphase, it increases muscular force during the deceleration subphase.

Accommodating elastic bands restrain the decrease in mechanical power output, velocity, and force during the deceleration subphase, which have middle to large effect sizes. However, they do not reduce the duration of the deceleration subphase. As the band resistance increased, the absolute duration of the deceleration subphase decreased. However, as the band resistance increased, the duration of the concentric phase also decreased. Therefore, the relative duration of the deceleration subphase was not significantly different according to band resistance. In contrast, as the band resistance increased, the mechanical power output during the deceleration subphase was increased. This result might be due to the characteristics of the elastic bands, which decrease the band tension in the first half of the concentric phase and increase it at the end of the concentric phase. The band resistance was applied at the middle point of the concentric phase and the total load of the barbell was above 56% 1RM at the latter middle point of the concentric phase. Thus, the mean force during the deceleration subphase increased as the band resistance increased. The band tension decreased the former half of the concentric phase, and the resultant total load of the barbell was below 56% 1RM. Therefore, the subjects were able to move the barbell more rapidly in the acceleration subphase until the point at which the deceleration subphase occurred, as compared with the no-band condition (B0). As a result, the subjects were able to start the deceleration subphase with high initial velocity, which leads to high mean velocity. BSQ with elastic bands significantly increase muscular force during the deceleration subphase. Above 20% band resistance of training load is recommended to increase the power output and 40% of training load is recommended to increase the force output during deceleration sub-phase.

Accommodating elastic bands also elicited greater mechanical power output during the acceleration subphase because the subjects were able to move the barbell more rapidly in the acceleration subphase. While the effect size observed was small (B20 [*d* = 0.28], B40 [*d* = 0.49], B60 [*d* = 0.65], and B80 [*d* = 0.71]), the force output during the acceleration subphase during BSQ with elastic bands (B20, B40, B60, and B80) was lesser than that with traditional free weight (B0). It has been reported in previous studies that the training effect of strength exercise is joint angle specific [6,19,20]. Therefore, it is possible that BSQ with elastic bands make it difficult to develop force output around the joint angle closer to the acceleration subphase. In short, if the training purpose is to increase forces in the respective angles of the acceleration subphase, then the elastic bands are not likely beneficial (Figure 3), although BSQ with elastic bands are effective for developing power during the acceleration subphase (Figure 6).

In a longitudinal study, the variable-resistance training group showed greater mechanical power output compared with the traditional free-weight training group (plates only) after training [8,21]. These results can be explained by the present finding (i.e., increase of mechanical power output during the acceleration and deceleration subphases). The purpose of using elastic bands during BSQ is to increase muscular force during the deceleration subphase. Meanwhile, the mean mechanical power, velocity, and force during the deceleration subphase were lesser than those of the acceleration subphase. Thus, the effect of accommodating elastic bands on BSQ may be significant but restrictive.

The study limitation was that the design was cross-sectional rather than longitudinal and the sample size was relatively small. Therefore, we were unable to make any conclusions about the effects of long-term resistance training on athletic performance. Significant differences were observed in some results. However, some of the results had no significant differences because of the small sample size. The power output equation might also be a limitation of this study. Lake et al. [22] reported that power output might be overestimated when barbell kinematics are used and kinematics of center of mass are recommended instead. However, this study investigated the results seen under different band conditions, and the trend of the results was unchanged, even if the actual measurement value of power output was overestimated. Therefore, we have adopted the LPT and force plates to calculate power output.

## 5. Conclusions

BSQ with elastic bands increases the mean power, velocity, and force during the deceleration subphase. However, these are lesser than those of the acceleration subphase. The force output during the acceleration subphase also slightly decreased when the band resistance increased. Therefore, VRE can substitute other power exercises (e.g., weightlifting movement, and ballistic exercise) in cases where the athlete is unable to perform other power exercises for various reasons (e.g., techniques, facilities, and equipment).

## Figures and Tables

**Table 1:** The equation of band resistance and R^2^ values.

Band Color	Regression Equation	R^2^
Purple	y = 20.802 ln(x) − 90.809	0.945
Red	y = 39.03 ln(x) − 171.9	0.983
Blue	y = 51.831 ln(x) − 227.85	0.989
Green	y = 90.823 ln(x) − 395.29	0.991

x = linear band deformation (cm); y = resistance applied (kg); and ln = natural log.

## References

[1] Haff G.G., Nimphius S. (2012). Training Principles for Power. Strength Cond. J..

[2] Loturco I., Kobal R., Kitamura K., Fernandes V., Moura N., Siqueira F., Cal Abad C.C., Pereira L.A. (2017). Predictive factors of elite sprint performance. J. Strength Cond. Res..

[3] Cormie P., McCaulley G.O., Triplett N.T., McBride J.M. (2007). Optimal loading for maximal power output during lower-body resistance exercises. Med. Sci. Sports Exerc..

[4] Lake J., Lauder M., Smith N., Shorter K. (2012). A comparison of ballistic and nonballistic lower-body resistance exercise and the methods used to identify their positive lifting phases. J. Appl. Biomech..

[5] Sanchez-Medina L., Perez C.E., Gonzalez-Badillo J.J. (2010). Importance of the propulsive phase in strength assessment. Int. J. Sports Med..

[6] Rhea M.R., Kenn J.G., Peterson M.D., Massey D., Simão R., Marin P.J., Favero M., Cardozo D., Krein D. (2016). Joint-angle specific strength adaptations influence improvements in power in highly trained athletes. Human Movement.

[7] Israetel M.A., McBride J.M., Nuzzo J.L., Skinner J.W., Dayne A.M. (2010). Kinetic and kinematic differences between squats performed with and without elastic bands. J. Strength Cond. Res..

[8] Joy J.M., Lowery R.P., Oliveira de Souza E., Wilson J.M. (2016). Elastic bands as a component of periodized resistance training. J. Strength Cond. Res..

[9] Kawamori N., Newton R.U. (2006). Velocity specificity of resistance training: actual movement velocity versus intention to move explosively. Strength Cond. J..

[10] Kilduff L.P., Bevan H., Owen N., Kingsley M.I.C., Bunce P., Bennett M., Cunningham D. (2007). Optimal loading for peak power output during the hang power clean in professional rugby players. Int. J. Sports Physiol. Perform..

[11] Suchomel T.J., Beckham G.K., Wright G.A. (2015). Effect of various loads on the force-time characteristics of the hang high pull. J. Strength Cond. Res..

[12] Suchomel T.J., Sole C.J. (2017). Force-time-curve comparison between weight-lifting derivatives. Int. J. Sports Physiol. Perform..

[13] Suchomel T.J., Wright G.A., Kernozek T.W., Kline D.E. (2014). Kinetic comparison of the power development between power clean variations. J. Strength Cond. Res..

[14] Wallace B.J., Winchester J.B., McGuigan M.R. (2006). Effects of elastic bands on force and power characteristics during the back squat exercise. J. Strength Cond. Res..

[15] Lake J.P., Mundy P.D., Comfort P., McMahon J.J., Suchomel T.J., Carden P. (2018). The effect of barbell load on vertical jump landing force-time characteristics. J. Strength Cond. Res..

[16] Haff G.G., Triplett N.T. (2015). Essentials of Strength Training and Conditioning 4th Edition.

[17] Shoepe T.C., Ramirez D.A., Almstedt H.C. (2010). Elastic band prediction equations for combined free-weight and elastic band bench presses and squats. J. Strength Cond. Res..

[18] Cohen J. (1998). Statistical Power Analysis for the Behavioral Sciences.

[19] Kubo K., Ohgo K., Takeishi R., Yoshinaga K., Tsunoda N., Kanehisa H., Fukunaga T. (2006). Effects of isometric training at different knee angles on the muscle-tendon complex in vivo. Scand. J. Med. Sci. Sports.

[20] Noorkoiv M., Nosaka K., Blazevich A.J. (2014). Neuromuscular adaptations associated with knee joint angle-specific force change. Med. Sci. Sports Exerc..

[21] Shoepe T.C., Ramirez D.A., Rovetti R.J., Kohler D.R., Almstedt H.C. (2011). The effects of 24 weeks of resistance training with simultaneous elastic and free weight loading on muscular performance of novice lifters. J. Hum. Kinet..

[22] Lake J.P., Lauder M.A., Smith N.A. (2012). Barbell kinematics should not be used to estimate power output applied to the Barbell-and-body system center of mass during lower-body resistance exercise. J. Strength Cond. Res..

